# Bioactive Foods and Medicinal Plants for Cardiovascular Complications of Type II Diabetes: Current Clinical Evidence and Future Perspectives

**DOI:** 10.1155/2021/6681540

**Published:** 2021-09-16

**Authors:** Roodabeh Bahramsoltani, Mohammad Hosein Farzaei, Mahboobe Ram, Shekoufeh Nikfar, Roja Rahimi

**Affiliations:** ^1^Department of Traditional Pharmacy, School of Persian Medicine, Tehran University of Medical Sciences, Tehran, Iran; ^2^PhytoPharmacology Interest Group (PPIG), Universal Scientific Education and Research Network (USERN), Tehran, Iran; ^3^Pharmaceutical Sciences Research Center, Health Institute, Kermanshah University of Medical Sciences, Kermanshah, Iran; ^4^Medical Biology Research Center, Kermanshah University of Medical Sciences, Kermanshah, Iran; ^5^Student Research Committee, Faculty of Pharmacy, Mashhad University of Medical Sciences, Mashhad, Iran; ^6^Evidence-Based Medicine Group, Pharmaceutical Sciences Research Center, Tehran University of Medical Sciences, Tehran, Iran; ^7^Department of Pharmacoeconomics and Pharmaceutical Administration, Faculty of Pharmacy, Tehran University of Medical Sciences, Tehran, Iran

## Abstract

Cardiovascular diseases (CVDs) are the main cause of mortality in type 2 diabetes mellitus (T2DM); however, not all patients are fully satisfied with the current available treatments. Medicinal plants have been globally investigated regarding their effect in CVD, yet the field is far from getting exhausted. The current paper aims to provide an evidence-based review on the clinically evaluated medicinal plants and their main therapeutic targets for the management of CVD in T2DM. Electronic databases including PubMed, Cochrane, Embase, Scopus, and Web of Science were searched from 2000 until November 2019, and related clinical studies were included. Lipid metabolism, glycemic status, systemic inflammation, blood pressure, endothelial function, oxidative stress, and anthropometric parameters are the key points regulated by medicinal plants in T2DM. Anti-inflammatory and antioxidant properties are the two most important mechanisms since inflammation and oxidative stress are the first steps triggering a domino of molecular pathological pathways leading to T2DM and, subsequently, CVD. Polyphenols with potent antioxidant and anti-inflammatory effects, essential oil-derived compounds with vasorelaxant properties, and fibers with demonstrated effects on obesity are the main categories of phytochemicals beneficial for CVD of T2DM. Some medicinal plants such as garlic (*Allium sativum*) and milk thistle (*Silybum marianum*) have strong evidences regarding their beneficial effects; however, others have low level of evidence which reveals the need for further clinical studies with larger sample sizes and longer follow-up periods to confirm the safety and efficacy of medicinal plants for the management of CVD in T2DM.

## 1. Introduction

Type 2 diabetes mellitus (T2DM) is a metabolic disorder accompanied with reduced glucose uptake, abnormal glucose metabolism, and insulin resistance which affects 1 in 11 adults [1]. Tracing the increasing rate of T2DM during the past decades shows its incidence and prevalence to be dramatically higher than the predicted values [2, 3].

T2DM is a principal risk factor for a broad spectrum of severe conditions amongst which the most important ones are cardiovascular diseases (CVDs) [4]. More than 80% of deaths in T2DM patients occur due to cardiovascular events which caused CVD to be the leading cause of mortality in these patients [5]. Based on the Framingham study, diabetic patients are two to six times more prone to myocardial infarction and heart failure [6]. A recent meta-analysis demonstrated higher risk of atrial fibrillation in diabetic patients [7]. Another meta-analysis revealed that diabetes significantly increases the risk of sudden cardiac death [8]. Also, hypertension is two times more prevalent in T2DM patients [9]. Diabetes is also an important risk factor for chronic heart failure [10]. Also, some cardiovascular drugs, such as statins, can increase the risk of diabetes[11]. Thus, there is a close relationship between T2DM and CVD.

Studies revealed that despite some beneficial effects of antihyperglycemic agents for the reduction of CVD risk [4], hyperglycemia is not the key concern in regard to CVD in T2DM patients [12]. In addition to glycemic control, specific management of CVD, e.g., regulation of lipid profile and HTN, is of a great importance [12]. In other words, antidiabetic agents such as metformin and glibenclamaide which only affect the blood glucose level does not have a considerable effect on CVD risk, whereas oral antidiabetics such as thiazolidinediones with insulin-sensitizing effects are more probable to reduce CVD risk [12, 13]. On the other hand, thiazolidinediones are associated with side effects such as higher risk of bone fracture due to the suppression of proosteoblastic pathways [14]. Other drug choices for the management of CVD risk in T2DM patients include antiplatelet agents and anticoagulants [4]; however, their long-term efficacy is still in doubt and more important, the adverse effects can be disturbing in long-term use, causing low patient adherence and compliance. So, scientists are globally seeking new options with better efficacy and fewer side effects for the management of CVD in T2DM.

Medicinal plants have an ancient history of use amongst people which is currently known as complementary and alternative therapies [15, 16]. Additionally, current scientific evidence supports the beneficial effects of herbal extracts and their isolated compounds in different types of CVD such as atherosclerosis [17], hypertension, and hyperlipidemia [18]. In diabetes-associated CVD, plant-derived natural supplements could play a positive role in preventing oxidative damage and inflammation [19] which are two basic mechanisms involved in the pathophysiology of T2DM-associated CVD. Thus, plants can be future candidates for the management of CVD in T2DM. The aim of the present study is to review controlled clinical trials on the cardiovascular effects of plants in T2DM patients.

## 2. Methods

### 2.1. Search Strategy

Electronic databases including Medline, Scopus, Embase, Web of Science, and Cochrane library were searched with the following search formula:

  “diabetes” [title/abstract/keyword] AND “plant” OR “extract” OR “herb” [all fields] AND “cardiovascular” OR “atherosclerosis” OR “hypertension” OR “hyperlipidemia” OR “dyslipidemia” [title/abstract/keyword]

Articles were collected from January 2000 until November 2019. Primary search results were screened by two independent investigators. No language restriction was considered.

### 2.2. Inclusion and Exclusion Criteria

Inclusion criteria were controlled clinical trials (using placebo or no intervention design) in which the effect of a plant in regard to a cardiovascular parameter (lipid profile, blood pressure, endothelial dysfunction, oxidative stress, or systemic inflammation) was evaluated in T2DM patients. Exclusion criteria were animal and cellular studies, human studies other than clinical trials (e.g., cohort studies and case reports), comparing the results of the herbal intervention with a standard drug (in case the standard drug was administered to both test and control groups, the study was included), choosing healthy individuals as control subjects, including type 1 diabetic patients or patients with obesity or metabolic syndrome without a diagnosis of T2DM, and administration of a mixture of herbal and nonherbal materials. Studies that assessed the effect of herbal mixtures were also excluded because the results of those studies cannot be attributed to any of the individual herbal extracts. Also, purified phytochemicals were excluded since the aim of this review is to only consider plants since they are better choices to be suggested as dietary interventions. Studies on the antidiabetic activity of plant extracts without considering a cardiovascular parameter were excluded, as well. References of the included articles were also checked to find further relevant studies.

Final included papers were screened to extract the scientific name of the plant (in case the scientific name was not mentioned, the most probable scientific name was written in the table with an asterisk sign), used part, dosage, study design, sample size (the number of patients who completed the study), duration of treatment, and outcomes. Jadad score was used to evaluate the quality of the studies [20].

## 3. Mechanisms of Plants Clinically Investigated to Control CVD in T2DM

**Figure 1:**
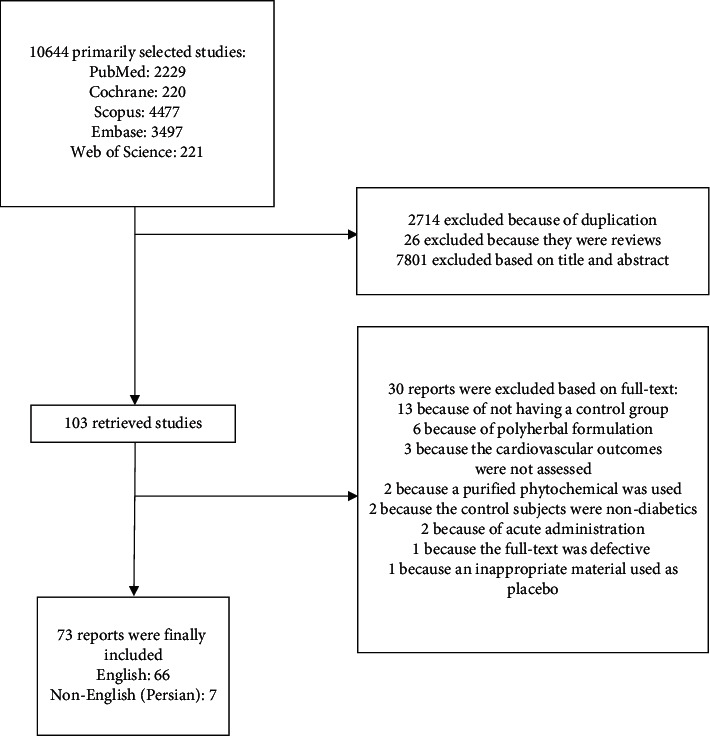
Study selection flow diagram.

**Figure 2:**
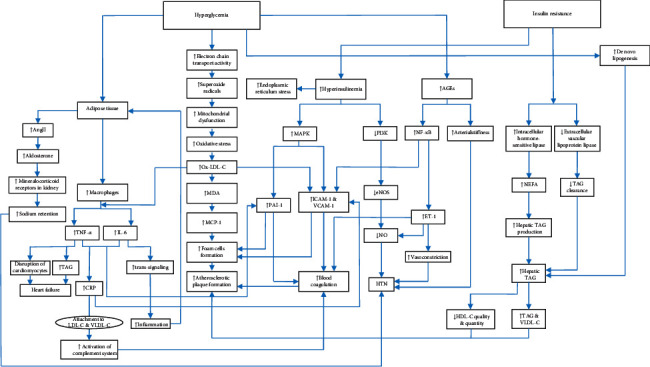
Therapeutic targets of bioactive foods and medicinal plants for the management of cardiovascular diseases in type 2 diabetic patients.

Among a total of 10644 primarily obtained papers, 73 were finally included.  Figure 1 shows the detailed study selection process. Final included papers are summarized in Table 1. Medicinal plants have demonstrated several benefits to control cardiovascular complications of T2DM via different mechanisms. The most important medicinal plants exerting each mechanism are discussed as follows.  Figure 2 shows a schema of the mechanisms affected by bioactive foods and plants to control CVD complications of T2DM.

### 3.1. Glycemic Profile

Despite the numerous large-scale clinical trials designed to clarify the relationship between blood sugar and CVD in T2DM patients, the problem is not yet completely solved since intensive blood glucose control has represented conflicting results [93]. Persistent hyperglycemia in diabetic patients leads to impaired angiogenesis which is, at least in part, related to abnormal glucose flux via the hexosamine biosynthetic pathway and participates in cardiovascular mortality [94]. Also, high level of blood glucose induces the production of advanced glycemic end-products (AGEs) which accumulate during the time and cause vascular complications of T2DM [95]. Blood insulin level is another key factor launching the cardiovascular complications of T2DM. It is demonstrated that type 1 diabetic patients (in whom the main contributor is the impaired insulin level) have also high susceptibility to CVD. Thus, regardless of the other factors, abnormal blood glucose and insulin level themselves can be triggers for cardiovascular events. Insulin acts as a double-edged sword in the pathogenesis of CVD. In healthy subjects, insulin secretion leads to the dual activation of the mitogen-activated protein kinase (MAPK) and the phosphatidylinositol 3-kinase (PI3K). While the former activates proatherogenic factors, the latter elevates nitric oxide (NO) production by endothelial nitric oxide synthase (eNOS) which causes a relaxing effect on the vascular smooth muscles and suppresses the proatherogenic and proinflammatory mediators in plasma. By contrast, during insulin resistance, the proatherogenic cascade is well activated, whereas the antiatherogenic pathway (PI3K) is not fully responsive [93].

Several medicinal plants have shown antihyperglycemic activity in clinical studies (Table 1). Milk thistle (*Silybum marianum* (L.) Gaertn.) is a well-known medicinal plant due to its ancient use as a hepatoprotective remedy; however, significant antioxidant properties of its flavonolignans made researchers reconsider its health benefits, and thus, it is now considered as an interesting option for the treatment of several chronic diseases involved with oxidative stress [96]. Six-week administration of milk thistle supplement to T2DM patients could significantly improve glycemic parameters via reduction of FBS, FPI, and insulin resistance which was evident from the homeostatic model assessment-insulin resistance (HOMA-IR) and quantitative insulin sensitivity check index (QUICKI) [21]. The supplement also improved lipid profile and inflammation/oxidative damage biomarkers [22]; thus, it can be suggested as a valuable herb to manage CVD risk in T2DM [97]. *Cynara scolymus* L. or the globe artichoke is a popular vegetable widely used in different parts of the world. In a study by Nazni et al., five different food products containing artichoke extract were prepared in order to choose the most pleasant form for a clinical trial on the effect of dietary artichoke on cardiovascular parameters in T2DM patients. Wheat biscuit enriched with artichoke extract was chosen as the most acceptable form and was administered in a placebo-controlled trial to T2DM patients for a period of 90 days. The preparation could significantly improve both fasting and postprandial blood sugar, as well as the lipid profile [23]. Another trial also assessed the effect of globe artichoke in T2DM patients; however, two months of treatment resulted in no significant effect on glycemic parameters and TAG in this study and only reduced TC and LDL-C [24]. It should be mentioned that, in addition to the shorter treatment period, the latter study used fiber-free extract, whereas the former study prepared the artichoke biscuits with the whole plant, containing fiber as well. This suggests the important role of dietary fibers in the clinical efficacy of artichoke.

### 3.2. Lipid Profile

Dyslipidemia is a common feature of T2DM with a prevalence of 37% to 56% [98]. The routine characteristics of diabetic dyslipidemia is hypertriglyceridemia in the form of elevated triacylglycerol-rich lipoproteins (TRLs), along with an increase in small dense low-density lipoprotein cholesterol (sdLDL-C) and a decrease in high-density lipoprotein cholesterol (HDL-C) [99]. TLRs are the result of fat digested from the foods as chylomicrons which contain apolipoprotein B48 (apoB48) or are released from the liver in the form of very-low-density lipoproteins (VLDL-C), containing apoB100 [100]. It seems that the reduction in CVD risk in diabetic patients via glycemic control is mostly due to the regulation of lipid profile rather than the glycemic profile itself [101]. Also, HDL-C is suggested to be an important factor which independently determines the risk of CVD in diabetic patients [102] since its quality and quantity are dysregulated even before the clinical diagnosis of diabetes [103]. In diabetic subjects, the level of intracellular hormone-sensitive lipase is augmented, while the extracellular vascular lipoprotein lipase is reduced due to insulin resistance or deficiency. The former results in the higher release of nonesterified fatty acids from adipose tissues which consequently increases hepatic production of triacylglycerol (TAG), whereas the latter causes reduced clearance of TAG from plasma; both finally result in the increased blood TAG level [103]. Increased blood sugar in T2DM elevates de novo lipogenesis from glucose [104]. Also, abnormal increase in insulin level along with insulin resistance causes disturbance in the production of hepatic sterols, endoplasmic reticulum stress, reduced apoB100 catabolism, and stimulation of VLDL-C production (especially VLDL_1_ which is larger and contains higher amount of TAG) that results in elevated blood VLDL-C level [99]. On the other hand, abnormal structure of HDL-C, as well as a decrease in its production, causes the antiatherogenic activity of these particles to be reduced compared with that of normal subjects [99].

Considering the abovementioned importance of lipid levels in T2DM, it is not far-fetched that most studies assessing the effect of medicinal plants in T2DM evaluate the effect of the treatments on the lipid profile of patients. Garlic (*Allium sativum* L.) is amongst the most evident medicinal plants to regulate the lipid profile. The bulb contains sulfated compounds such as S-allyl cysteine, allicin, and alliin. When chopped, alliinase in garlic turns alliin to allicin [105]. Garlic is suggested to have an antihypertrophic effect on the heart via the elevation of hydrogen sulfide (H_2_S) and NO [106]. Alliin has shown an inhibitory effect on 3‐hydroxy 3‐methyl glutaryl coenzyme A (HMG-CoA) reductase activity which is the same mechanism as statins [106]. Garlic also showed antihypertensive activity [107] as well as an inhibitory effect on adenosine deaminase (ADA) activity, an enzyme possibly involved in insulin resistance [108]. Some studies reported significant effect of garlic supplement with a dose of 500 mg/day [108] or 300 mg, twice daily [25, 26] in the regulation of lipid profile; whereas some others such as the study of Atkin et al. reported no such effect even with a higher administered dose [27]. This might be due to the different design of the two studies as the patients in the latter study received conventional antidiabetics, as well. Also, the latter study assessed the effect of garlic for a shorter period of time which shows the need for longer treatment to achieve the therapeutic effect. Overall, garlic can be considered as an important dietary intervention to manage a series of cardiovascular complications such as HTN, hyperlipidemia, and atherosclerosis [109] which is also reported in T2DM patients; however, future studies are needed to determine the optimum dose and dosage form to achieve a satisfying therapeutic outcome. Ginger rhizome (*Zingiber officinale* Roscoe) is another medicinal plant which is also used as a popular spice in cooking. Three months of supplementation with ginger in T2DM patients could significantly decrease LDL-C and TAG and improve HDL-C. Ginger could also significantly reduce fasting plasma insulin and HOMA-IR, showing an overall improvement in lipid profile, as well as insulin resistance [28]. These results regarding the insulin resistance and TAG were also confirmed in another study by Arablou et al.; however, the reported data on the LDL-C, HDL-C, and FBS were somehow controversial [29]. While the former study reported a nonsignificant effect on blood glucose, HDL-C, and HbA1c, the latter study showed a significant decrease in FBS and HbA1c, as well as an increase in HDL-C which might be due to the longer period of supplementation. Ginger could also improve the biomarkers of oxidative damage and inflammation [30], suggesting the plant as a multipotential choice to manage CVD in T2DM. Gingerol and shogaol are two main active ingredients of the plant responsible for several pharmacological activities such as anti-inflammatory and vasorelaxant effects via the inhibition of prostaglandin and leukotriene synthesis. Also, ginger has demonstrated antiplatelet activity in animal studies which is another beneficial mechanism in CVD [110]; however, it should be noted that, in patients taking conventional antiplatelet agents, high doses of ginger may show a synergistic pharmacodynamic herb-drug interaction, thus, should be taken with caution.

### 3.3. Systemic Inflammation

The presence of inflammation in T2DM has been demonstrated years ago through cohort studies that revealed higher incidence of the disease in patients with higher level of acute-phase proteins such as C-reactive protein (CRP) and proinflammatory cytokines such as interleukin-6 (IL-6) in comparison to the subjects with normal values of these markers [111]. The increased level of inflammatory markers in T2DM is suggested to be mostly due to a general inflammatory status involving the whole body's immune system rather than a local inflammation of pancreas. Adipose tissue, abnormally hosting a large number of macrophages in obese subjects which produce tumor necrosis factor (TNF), and the liver, in which TNF-*α*- and IL-6-induced CRP is produced, seem to be the main participants in this process [112]. Inflammation is a common characteristic of T2DM and CVD. Overproduction of TNF-*α* and proinflammatory ILs results in cardiovascular events such as atherosclerosis. The importance of CRP in the prediction of CVD risk in T2DM is as high as LDL-C and HDL-C. On one hand, CRP attaches to LDL-C and VLDL-C particles and triggers blood coagulation via the activation of the complement system. On the other hand, it stimulates the production of soluble adhesion molecules, facilitating the formation of foam cells, and makes the endothelium of blood vessels prone to atherosclerotic plaque formation [113]. The deep involvement of CRP in cardiovascular events, along with its long half-life suggests this marker as an independent factor predicting CVD risk in T2DM [114]. Elevated level of TNF-*α* is also associated with increased risk of heart failure via the elevation of TAG, possibly due to stimulating VLDL-C production and disruption of cardiomyocytes [113]. In contrast, IL-6 seems to have a pleiotropic role in CVD which in not yet completely clarified; however, the mechanisms are somehow explained after the discovery of its two underlying signaling pathways, i.e., classic signaling and trans-signaling. While the activation of the former pathway results in anti-inflammatory effects and regulates the metabolism, the latter exacerbates inflammation during pathological conditions. The ratio of IL-6, soluble IL-6 receptor, and glycoprotein 130 (gp 130, a common signal transducer in the IL-6 family) determines the final results of IL-6 activation [115]. It is also demonstrated that IL-6 and TNF-*α* interaction plays an important role in endothelial dysfunction in the animal model of diabetes [116].

Some medicinal plants have been reported to be effective on the serum level of inflammatory markers in T2DM patients. *Panax ginseng* C.A. Mey. (Chinese ginseng or Korean ginseng) is a globally well-known medicinal plant with a wide spectrum of indications in traditional Chinese medicine (TCM). Administration of ginseng supplement to diabetic patients over a period of eight weeks significantly reduced the serum level of IL-6 and CRP in comparison to both baseline values and the placebo group [31]. One of the main classes of active ingredients of ginseng are triterpene structures called ginsenoside, several of which have demonstrated anti-inflammatory properties via the modulation of IL-6 production [117], and their beneficial effects in CVD are demonstrated in preclinical studies [118]. Another medicinal plant effective on the level of inflammatory markers is stinging nettle (*Urtica dioica* L.) which decreased IL-6 and CRP vs. placebo and TNF-*α* compared with baseline levels [32]. Another trial also demonstrated a higher level of NO in patients treated with nettle extract in comparison to placebo [33]. Based on the preclinical investigations, the antidiabetic effect of stinging nettle is attributed to its phenolic compounds and seems to be due to cytoprotective effects on the pancreas, *α*-glucosidase and *α*-amylase inhibition, and modulation of glucose transporter type 4 [119]; however, the exact subcellular mechanism of the plant in the modulation of inflammatory mediators needs to be further investigated.

### 3.4. Oxidative Stress

Oxidative stress is an inseparable part of T2DM and is closely related to the cardiovascular complications of this disease [120, 121]. High blood sugar causes oxidative stress via the elevation of AGEs, glucose auto-oxidation, and hexosamine and polyol pathways induction, as well as mitochondrial damage due to overactivation of the electron chain transport system, resulting in the overproduction of superoxide radicals which are naturally deactivated by superoxide dismutase (SOD) enzyme [95, 122]; however, the excessively produced radicals directly destruct mitochondrial DNA. This damage causes a series of events leading to mitochondrial dysfunction and abnormal cellular energy expenditure, further inducing oxidative stress [95]. Oxidative stress in diabetic patients causes LDL-C particles turn into an oxidized form (Ox-LDL-C), evident from the serum level of malondialdehyde (MDA, a byproduct of lipid peroxidation) that consequently stimulates the production of monocyte chemoattractant protein-1 (MCP-1), a trigger for the formation of foam cells [121]. This oxidation process is partially prevented by paraoxonase-1 (PON-1), an esterase linked with apo A-1 of HDL-C particles, thus, plays a protective role against the development of atherosclerotic plaque formation [123]. PON-1 is also suggested to be a reliable marker for the prediction of atherosclerosis risk in T2DM [124]. Glutahtoine (GSH) is another part of the endogenous antioxidant defense mechanism which is altered in T2DM. It is demonstrated that the GSH level of erythrocytes and its absolute synthesis rate in diabetic patients are significantly lower than those of normal subjects, possibly due to nonglycemic mechanisms [125].

*Punica granatum* L. (pomegranate) is native to the Mediterranean region, especially Iran. The fruit juice is a rich source of two polyphenol subcategories, namely, anthocyanins and ellagitannins, with potent antioxidant and anti-inflammatory properties, as well as previously demonstrated beneficial effects in cardiovascular problems [126]. In a randomized, single-blind clinical trial, T2DM patients received a daily amount of 200 ml pomegranate juice for 6 weeks. Compared to the untreated group, pomegranate could significantly improve antioxidant status via the elevation of PON-1 and decrease in Ox-LDL-C, as well as its specific antibodies [34]. Shidfar et al. reported the significant effect of cranberry, another anthocyanin-rich fruit, toward the prevention of oxidative damage in T2DM via the improvement of PON-1 [35]. Grape seed extract, rich in proanthocyanidins, has demonstrated antioxidant properties in T2DM which was evident from the increased level of GSH [36]. It seems that anthocyanin-rich fruits are a reliable source of antioxidant components, especially in regard to improvement in PON-1 activity , thus, are clinically valuable to manage chronic oxidative damage in T2DM [127].

### 3.5. Blood Pressure

Hypertension (HTN) in T2DM is linked with a dramatic raise in the incidence of CVD so that a 10 mmHg decrease in systolic blood pressure (SBP) is accompanied with 11% and 13% lower risk of myocardial infarction and microvascular complications, respectively [128]. Also, T2DM patients with diastolic blood pressure (DBP) lower than 80 mmHg showed a lower risk of stroke and mortality compared with those with a DBP of 90 mmHg [129]. Chronic inflammation in T2DM causes endothelial dysfunction, evident from the reduction of flow-mediated dilatation (FMD), makeing an imbalance between endogenous vasodilators such as NO and vasoconstrictors such as endothelin-1 (ET-1). Also, inflammation and oxidative stress stimulate the production of antgiotensin II in adipose tissue which results in increased aldosterone, the activation of mineralocorticoid receptors in the kidney, sodium retention, and consequently, HTN [130, 131]. Furthermore, the production of AGEs due to high blood sugar causes stiffness of the arterial wall, further exacerbating HTN [9].

American ginseng (*Panax quinquefolius* L.) is grown in the North American continent. In a clinical trial in T2DM patients with well-controlled essential HTN, American ginseng ethanolic extract, standardized based on 10% of total ginsenosides, was administered to evaluate the vascular effects. The results showed a significantly lower SBP in the active group compared with placebo. Additionally, the augmentation index which is a noninvasive method for the evaluation of arterial stiffness was significantly reduced in patients treated with ginseng [37]. Preclinical studies suggest the inhibitory effect on glucotoxicity and endothelial dysfunction [132] and vascular smooth muscle cell proliferation [133] to be the possible vasculoprotective mechanisms of American ginseng. Barberry is another plant that demonstrated beneficial effects on both SBP and DBP in T2DM patients compared with the no intervention group [38]. This effect may be partly mediated by berberine, an alkaloid in barberry fruit, with previously demonstrated antihypertensive and vasodilatory properties in preclinical studies [134, 135]. Barberry fruit was also effective in reducing blood glucose and insulin, as well as lipid profile, which is also attributed to berberine [39]. Barberry fruit contains anthocyanins, a group of polyphenols, which may also contribute to the health benefits of the fruit; however, a clinical trial on the barberry stems containing 2.23% berberine as the major alkaloid [40] (but a possibly lower anthocyanin content) in T2DM patients further confirms the significant role of this alkaloid in glycemic and cardiovascular effects of barberry.

### 3.6. Endothelial Function

Endothelial dysfunction is suggested as an independent predictor of T2DM development risk. Adhesion molecules including intracellular adhesion molecule (ICAM-1) and vascular adhesion molecule-1 (VCAM-1) are produced as a result of endothelial dysfunction. They are demonstrated to get increased years before the diagnosis of T2DM, and thus, they are not only the markers of vascular damage but also predict the occurrence of T2DM [136, 137]. High production of AGEs in T2DM activates nuclear factor-*κ*B (NF-*κ*B) which consequently stimulates gene expression of several factors, including ICAM-1, VCAM-1, and ET-1 [138]. Enhanced activity of ET-1 negatively affects the cardiovascular status of diabetic patients due to vasoconstrictor and procoagulant activities of ET-1, as well as a reduction in NO-mediated vasodilation [139]. On the other hand, proinflammatory cytokines such as TNF-*α* and IL-6 trigger the coagulation process via the elevation of von Willebrand Factor (VWF) and plasminogen activator inhibitor-1 (PAI-1), resulting in a procoagulant state in the impaired endothelium. Furthermore, increased ox-LDL-C induces the production of proinflammatory cytokines, MCP-1, and adhesion molecules, all of which facilitate the process of atherosclerosis [138].

In a pilot-controlled trial, *Salvia miltiorrhiza* Bunge. was assessed in T2DM patients with coronary heart disease. The plant is commonly known as danshen and is a popular natural remedy for CVD in TCM. Two-month supplementation with danshen extract significantly decreased the level of VCAM-1 and VWF, demonstrating an improvement in endothelial function [41]. Salvianolic acids and diterpene structures called tanshinones are two major categories of danshen phytochemicals, both of which participate in beneficial effects of the plant in CVD [140]. Another study evaluated the vasculoprotective effect of ginkgo (*Ginkgo biloba* L.) on T2DM patients with nephropathy. Ginkgo, known as a living fossil, contains a mixture of flavonoids (ginkgoflavone glycosides) and diterpenes (ginkgolides and bilobalide) as the main active ingredients [141]. The plant has shown an inhibitory effect on ox-LDL-C-induced endothelial dysfunction in human umbilical vein endothelial cells [141]. Supplementation with ginkgo leaf extract could significantly attenuate the abnormally increased VWF and elevate NO level. Also, there were no significant differences in FBS, showing that the plant can be coadministered along with conventional antidiabetics without increasing the risk for hypoglycemia [42].

### 3.7. Anthropometric Parameters

The relationship between obesity and T2DM has been demonstrated in several studies. Recently published results of the ACCORD (Action to Control Cardiovascular Risk in Diabetes) study showed that T2DM patients with a body mass index (BMI) higher than 40 have the highest risk of nonfatal myocardial infarction and cardiac death [142]. Adipocytes secrete nonesterified fatty acids and hormones such as adiponectin and leptin which affect metabolism and insulin sensitivity. The imbalance between the release of these agents results in *β*-cell dysfunction and insulin resistance [143]. Molecular assessments also revealed that the gene expression of AGE ligands, NF-*κ*B, and PI3K is dysregulated in obesity and T2DM [144]. Although obesity, in general, is known as a risk factor for T2DM, the distribution of body fat is a more important indicator of insulin resistance. It is suggested that waist circumference is the determinant of abdominal obesity and can be used to predict the risk of T2DM and CVD [145, 146]. Aside direct exacerbating effects of T2DM on CVD, the coexistence of obesity in these patients further worsens the condition since an accumulating body of evidence introduces obesity as one of the prime suspects of CVD [147].

Guar gum is a product of *Cyamopsis tetragonoloba* (L.) Taub. seeds and is a water-soluble polymer of galactose and mannose usually used in a partially hydrolyzed form due to the unpleasantly high viscosity of the original form [148]. Six-week supplementation with daily 10 g dose of guar gum could significantly reduce HbA1c and serum *trans*-fatty acids compared with baseline values. The plant could also significantly reduce waist circumference; however, no such effect was observed for body weight (BW) [43]. Previous studies have shown beneficial effects of guar gum in weight loss due to the gel-forming properties and decreasing gastric emptying speed, as well as antiappetite activity [148]; thus, lack of the slimming effect in this trial might be due to the short study period or low administered dose. Another plant with high content of water-soluble fiber which is assessed in regard to CVD risk factors in T2DM is *Avena nuda* L., commonly known as naked oat. In this dietary intervention, patients were instructed to replace one of the main foods of their regimen with a product providing 50 or 100 g of naked oat. One month of naked oat intake decreased BW, BMI, and waist circumference in comparison to the control group in a dose-dependent manner. Both lipid and glycemic profile were also improved during this intervention [44]. It can be inferred from the two studies that water-soluble fibers are one of the important categories of plant-based products able to help weight loss and glycemic/lipid profile regulation in T2DM patients; however, since they are mostly effective in high doses, they are suggested to be administered as dietary interventions.

## 4. Safety

Most of the studies included in this review reported no significant difference between the frequency of adverse effects in the active and passive groups, especially in case of dietary interventions, showing that the preparations were well tolerated. Most of the reported adverse effects were limited to transient low-grade gastrointestinal complications which disappeared after a while or by dose reduction. Also, some studies assessed the safety by measuring the biomarkers of hepatic and renal toxicity including aspartate transaminase (AST), alanine transaminase (ALT), and alkaline phosphatase (ALP), as well as blood urea and creatinine, respectively. Most studies reported no significant change in the evaluated biomarkers except within the normal range. Taken together, herbal interventions are generally safe in regard to the adverse effects; however, some other considerations such as the possibility of herb-drug interactions via pharmacokinetic or pharmacodynamic interactions with the conventional antihyperglycemic and cardiovascular drugs are possible which should be taken into account [149, 150]. As an example, ginseng and garlic can increase prothrombin time, and if being administered to patients under treatment with aspirin or warfarin, the synergistic effect can result in abnormal bleeding [151]. Also, mucilaginous herbal materials, such as different gums, as well as resinous compounds, can delay/decrease the oral absorption of concomitantly used conventional drugs and consequently affect their pharmacokinetics; thus, it is recommended to take such herbal supplements with a proper time interval with conventional drugs [152].

## 5. Discussion

The current paper reviewed recent advances regarding the effectiveness of plants as dietary interventions for the management of cardiovascular complications in T2DM. It is well understood that any individual plant can act via several mechanisms which suggest it as a multifaceted approach to target different pathways involved in the pathogenesis of CVD in T2DM.

Two of the most important mechanisms by which several medicinal plants could improve cardiovascular outcomes of diabetic patients are antioxidant and anti-inflammatory properties. Oxidative damage and inflammation can trigger each other due to a negative feedback loop so that the free radicals can induce inflammation, and inflammatory mediators increase oxidative stress in a chronic pathological condition such as T2DM. Discovering detailed cellular pathways which participate in the pathogenesis of cardiovascular damage is the Rosetta stone for decoding the fundamental therapeutic targets in CVD of T2DM patients. Plants are rich sources of secondary metabolites preventing the free radicals produced due to the oxidative damage in different tissues which is now demonstrated in clinical studies (Table 1). Although most of the included trials have focused on the lipid and glycemic profile which are classic outcomes to be assessed in CVD, recent studies have tried to also measure oxidative damage and inflammation biomarkers due to the growing evidence supporting the involvement of these two mechanisms in the primary stages of T2DM.

The most important category of plant-derived secondary metabolites with antioxidant activities are polyphenols (Table 2). Polyphenols comprise several subcategories including flavonoids, anthocyanins, lignans, and phenolic acid, each of which have numerous number of studies supporting their antioxidant and anti-inflammatory activities [17]. Curcumin, resveratrol, quercetin, and epigallocatechin gallate (EGCG) which are today widely used as antioxidant supplements all belong to different classes of polyphenols.

Another important category of phytochemicals are essential oil-derived compounds such as small terpenes and terpene alcohols. These compounds have demonstrated relaxing effects on the muscle cells, thus, can improve HTN via vasorelaxant effects on the smooth muscles of blood vessels [153, 154].

Fiber-containing plants are also significantly effective in controlling CVD risk factors in T2DM patients by slowing the absorption of dietary fats and improvement of anthropometric parameters. One of the positive points regarding this category of phytochemicals is that they are usually tasteless compounds, so they can be prepared as different enriched food products without affecting the original taste, providing higher patient compliance.

In addition to fibers, other herbal materials can also be provided as enriched foods, e.g., beverages, breads, biscuits, corn flakes, or other types of usually taken snakes. Plants with active ingredients resistant to heat can be added to baked products such as the preparation used in the study of Nazni et al. [23]. Anthocyanin-rich fruits such as barberry, pomegranate, and cranberry can be prepared as cold beverages with natural bright colors which can be easily taken as a daily routine. Such preparations with medicinal properties without having the appearance of a typical medicine are more welcomed by patients since diabetic patients usually receive lots of conventional medicines and thus the addition of a pack of healthy biscuits or a glass of natural juice would be more pleasant than another series of capsules/tablets.

Some of the abovementioned medicinal plants have strong evidence to support their beneficial effects in CVD of T2DM; however, some others have only limited data regarding their safety and efficacy. Also, some medicinal plants have controversial data obtained in different clinical trials. One of the obvious reasons explaining these controversial results is the difference between study duration and sample size. Some biomarkers need a specific minimal time to be changed as the results of an intervention which is not considered in some trials. For instance, HbA1c, as the gold standard of glycemic control, needs a 1.5 to 2 months of time to show the results of the intervention, whereas some clinical trials are designed for a shorter period of time, resulting in nonsignificant inter/intragroup difference which may be wrongly considered as a negative result [155].

The dosage and formulations are other important factors affecting the result of a study. Some phytochemicals such as curcuminoids in turmeric have highly lipophilic structures, causing a poor oral bioavailability; thus, bioavailability-enhanced formulations provide higher serum concentrations of the active ingredients and consequently better clinical outcomes. Another important reason which usually remains undiscussed is the different baseline characteristics of the selected patients. Several factors including the onset of T2DM, duration of previous pharmacotherapy to control the disease, and patient adherence to the prescribed medicines, as well as genetic factors such as the race and family history of T2DM, can cause different treatment responses to the same therapeutic interventions and should be carefully considered when comparing the outcomes of different trials on the same plant. Thus, negative results obtained in patients with a long history of T2DM, usually evident from the level of HbA1c, do not necessarily mean that the intervention cannot be effective in newly diagnosed patients [155].

The amount of main active ingredients in the herbal preparation is another factor which may affect the final results. As it has been discussed in several previous literature [156, 157], the amount of phytochemicals depends on several factors such as the time of harvest, storage condition, and extraction method. Lack of optimization of the production procedure results in nonuniform preparations which can affect the clinical outcome. The best way to solve this problem is to consider an optimum standardization procedure based on the major components of the plant. This can be based on a precise technique such as high-performance liquid chromatography (HPLC) or an easier but faster method such as colorimetric spectroscopic techniques. Announcing the level of main active compounds of the herbal preparations in the clinical trials can help comparing the quality of preparations in different studies and gives a more reliable judgement regarding the obtained results.

One of the limitations of our study is that the types of interventions were diverse; thus, the results cannot be subjected to statistical analysis since they do not fulfill the essential criteria for systematic reviews and meta-analysis. However, the collected information is valuable as a comprehensive interpretation of the current clinical evidence on the management of CVD in T2DM by medicinal plants. Another limitation is that only placebo-controlled trials were included in this paper. These criteria were considered in order to include fewer studies with more homogeneity so that different interventions can be compared with each other. There are several uncontrolled studies or studies on the comparison of an herbal intervention with a positive control which can also provide valuable data and may be the subject of future review articles on the same topic.

## 6. Conclusions

Overall, several plants are capable of improving the cardiovascular complications of T2DM and can be suggested as complementary therapies or dietary interventions along with conventional medicines. Active components of the clinically effective medicinal plants can also be used as new backbones to develop semisynthetic structures with higher potency and controlled profile of adverse effects for CVD. Further preclinical studies to clarify the exact cellular and subcellular mechanisms of these natural products, as well as well-designed clinical studies, are necessary to confirm the safety and efficacy of plant-based therapies for the management of CVD in T2DM.

## Figures and Tables

**Table 1:** Clinical studies on the use of medicinal plants for the management of cardiovascular complications in diabetic patients.

Plant/part	Family	Common name	Dosage	Design	Duration	Jadad score	Concomitant therapy	Outcomes	Reference
*Acca sellowiana* (O. Berg) Burret/fruit	Myrtaceae	Feijoa	150 mg	Randomized, double-blind, placebo-controlled trial on diabetic patients treated with the plant (20) or placebo (14)	3 m	2	Oral antidiabetics and/or insulin	Vs. placebo: ↓FBS and HbA1c, ↓LDL-C, TC, and TAG, ↑HDL-C, and ↓SBP and DBP	[45]
*Allium sativum* L./bulb	Amaryllidaceae	Garlic	250 mg, BD	Randomized, open-label, prospective, comparative trial on obese diabetic patients treated with metformin (30) or metformin + plant (30)	3 m	1	ND	Vs. baseline and placebo: ↓FBS and GTT, ↓TC, LDL-C, and TAG, ↑HDL-C, ↓ADA and CRP, and ↓HbA1c vs. metformin alone, but not vs. baseline	[13]
*Allium sativum* L./bulb	Amaryllidaceae	Garlic	300 mg, BD	Placebo-controlled trial on diabetic patients treated with the plant (10) or placebo (10) as monotherapy or the plant (20) or placebo (20) concomitant with oral antidiabetics	1 m	3	None in the monotherapy groups, oral antidiabetics in the combination-therapy groups	Monotherapy: vs. baseline: ↓TAG and fructosamine; vs. placebo: ↓FBS and fructosamine, combination therapy: vs. baseline: ↓TAG and fructosamine and ↓15% in the dosage of oral antidiabetics, and vs. placebo: ↓fructosamine and no significant change in TC, LDL-C, and HDL-C	[46]
*Allium sativum* L./bulb	Amaryllidaceae	Garlic	300 mg, TDS	Randomized, single-blind, placebo-controlled trial on diabetic patients treated with the plant (30) or placebo (30)	6 m	2	Metformin	Vs. placebo: ↓TC, LDL-C, and TAG and ↑HDL-C	[26]
*Allium sativum* L./bulb	Amaryllidaceae	Garlic	300 mg, BD	Randomized, single-blind, placebo controlled in dyslipidemic diabetic patients treated with the plant (35) or placebo (35)	3 m	2	ND	Vs. placebo: ↓TC and LDL-C and ↑HDL-C	[25]
*Allium sativum* L./bulb	Amaryllidaceae	Garlic	300 mg, QID	Randomized, double-blind, placebo-controlled, crossover trial on 26 diabetic patients with high risk for cardiovascular diseases	1 m	3	Metformin, statins, ASA, and ACE inhibitors	Vs. placebo: no significant change in BP, lipid profile, anthropometric parameters, oxidative stress, and inflammation biomarkers	[27]
*Aloe vera* (L.) Burm.f./leaf gel	Xanthorrhoeaceae	Aloe	300 mg, BD	Randomized, double-blind, placebo-controlled trial on diabetic hypercholesterolemic patients treated with the plant (30) or placebo (30)	2 m	5	Metformin and glyburide	Vs. baseline and placebo: ↓FBS and HbA1c and ↓TC and LDL-C and no significant change in HDL-C and TAG	[47]
*Aloe vera* (L.) Burm.f./leaf gel	Xanthorrhoeaceae	Aloe	1 g daily	Randomized, double-blind, placebo-controlled trial on diabetic patients treated with the plant (21) or placebo (22)	2 m	5	Oral antidiabetics	Vs. baseline: no significant change in glycemic parameters and lipid profile	[48]
*Avena nuda* L./seed	Poaceae	Naked oat	50 or 100 g/ day	Randomized, single-blind, controlled trial on diabetic hyperlipidemic patients treated with usual care (63), diet intervention (61), 50 g of the plant (65), or 100 g of the plant (71)	1 m	3	Oral antidiabetics and/or insulin	Vs. control: ↓FBS, post prandial glucose, HbA1c, and HOMA-IR ↓TC, LDL-C, and TAG, ↓BW, BMI, and WC (only with high dose), and ↑HDL-C	[44]
*Berberis aristata* DC^*∗*^/stem bark	Berberidaceae	Indian barberry	0.75 g or 1.5 g, BD	Randomized, open, controlled trial on diabetic patients treated with the low-dose of the plant (30) and high-dose of the plant (30) or left untreated (30)	9 m	2	Oral antidiabetics and antihyperlipidemics	Vs. control: ↓FBS, ↓TAG and LDL-C, ↑HDL-C, and no significant change in TC	[40]
*Berberis vulgaris* L./fruit	Berberidaceae	Barberry	1 g, TDS	Randomized, double-blind, placebo-controlled trial on diabetic patients treated with the plant (21) or placebo (21)	3 m	4	Oral antidiabetics	Vs. placebo: ↓FBS and FPI, ↓TAG, TC, LDL-C, and apo-B, ↑TAOS, and no significant change in HbA1c, homocysteine, and HDL-C	[39]
*Berberis* sp.^*∗*^/fruit	Berberidaceae	Barberry	200 ml, daily	Randomized, double-blind, controlled trial on diabetic patients treated with the plant (23) or left untreated (23)	2 m	3	Oral antidiabetics	Vs. control: ↓FBS, ↓TC, ↑PON-1, ↓SBP and DBP, ↓BW and BMI, and no significant change in HbA1c, TAG, LDL-C, HDL-C, apo A1, and apo B100	[38]
*Boswellia serata* Roxb. ex Colebr./gum resin	Burseraceae	Olibanum	400 mg, BD	Randomized, double-blind, placebo-controlled trial on diabetic patients treated with the plant (37) or placebo (34)	3 m	4	Metformin	Vs. placebo: ↓FBS, HbA1c, and FPI, ↓TC and TAG, and no significant change in HDL-C	[49]
*Brassica napus* L./seed oil	Brassicaceae	Canola	31 g canola oil/2000 kcal	Randomized, controlled trial on diabetic patients treated with canola-enriched bread (55) or wheat bread (64)	3 m	3	Oral antidiabetics	Vs. control: ↓HbA1c, ↓TC, LDL-C, TAG, TC/HDL-C, and LDL-C/HDL-C, ↓Framingham CVD risk score, and no significant change in BP, HR, BW, and WC	[50]
*Brassica oleracea* L./sprout	Brassicaceae	Broccoli	5 or 10 g/day	Randomized, double-blind, placebo-controlled trial on diabetic patients treated with the low-dose plant (26), high-dose plant (23), or placebo (23)	1 m	5	Oral antidiabetics	Vs. baseline: ↓FBS and ↓TC and LDL-C; high-dose vs. low-dose and placebo: no significant effect on FBS, TC, LDL-C, ↓TAG, ox-LDL-C/LDL-C, AIP, and ↑HDL-C	[51]
*Carica papaya* L./fermented fruit	Caricaceae	Papaya	3 g, BD	Randomized, controlled trial on total of 101 diabetic patients	4 m (2 w of washout at the end)	3	—	Vs. control: ↓SBP, ↑TAG, ↑TAOS, and no significant change in glycemic parameters, TC, LDL-C, and HDL-C	[52]
*Cinnamomum cassia* (L.) J.Presl/bark	Lauraceae	Cinnamon	500 mg, 2, 6, or 12 caps/day	Randomized, placebo-controlled trial on diabetic patients in six groups treated with two (10), six (10), and twelve (10) plant capsules or two (10), six (10), or twelve (10) placebo capsules	40 days of treatment and 20 days of follow-up	1	Oral antidiabetics	Vs. baseline: ↓FBS, ↓TC, TAG, and LDL-C, and no significant change in HDL-C	[53]
*Cinnamomum cassia* (L.) J.Presl/bark	Lauraceae	Cinnamon	400 mg, TDS	Randomized, placebo-controlled trial on diabetic patients treated with the plant (29) or placebo (30)	3 m	2	Oral antidiabetics	Vs. placebo: no significant change in glycemic profile, lipid profile, and anthropometric parameters	[54]
*Cinnamomum zeylanicum* Blume/bark	Lauraceae	Cinnamon	1 g, TDS	Randomized, double-blind, placebo-controlled trial on diabetic patients treated with the plant (20) or placebo (19)	2 m	4	Metformin	Vs. placebo and baseline: no significant change in glycemic profile, MDA, and TAOS	[55]
*Crataegus laevigata* (Poir.) DC./flower and leaf	Rosaceae	Hawthorn	400 mg, TDS	Randomized, double-blind, placebo-controlled trial on diabetic dyslipidemic patients treated with the plant (24) or placebo (21)	6 m	4	ASA, statins, and antihypertensives	Vs. baseline: ↓TC, LDL-C, and non-HDL-C and no significant change in TAG and HDL-C; vs. placebo: ↓neutrophil elastase and no significant change in lipid profile, CRP, and MDA	[56]
*Crataegus laevigata* (Poir.) DC.^*∗*^/fruit	Rosaceae	Hawthorn	600 mg, BD	Randomized, double-blind, placebo-controlled trial on diabetic hypertensive patients treated with the plant (39) or placebo (40)	4 m	5	Oral antidiabetics and antihypertensives	Vs. placebo: ↓DBP and no significant change in SBP, FBS, HbA1c, and fructosamine	[57]
*Crocus sativus* L./stigma	Iridaceae	Saffron	15 mg, BD	Randomized, double-blind, placebo-controlled trial on diabetic patients treated with the plant (32) or placebo (32)	3 m	5	Oral antidiabetics	Vs. baseline: ↓FPG, HbA1c, TC, LDL‐C, and LDL-C/HDL-C ratio; vs. placebo: ↓FPG, TC, LDL‐C, and LDL-C/HDL-C ratio and no significant change in anthropometric parameters, TAG, and HDL-C	[58]
*Cucurbita ficifolia* Bouché/fruit	Cucurbitaceae	Pumpkin	100 g daily	Randomized, controlled trial on diabetic patients treated with the plant (20) or left untreated (20)	2 m	2	Metformin and glibenclamide	Vs. baseline: ↓FBS and HbA1c, ↓SBP and DBP, and ↓LDL-C and CRP; vs. control: ↓FBS, HbA1c and ↓SBP and DBP	[59]
*Curcuma longa* L./rhizome	Zingiberaceae	Turmeric	750 mg, BD	Randomized, double-blind, placebo-controlled trial on diabetic patients treated with the plant (99) or placebo (100)	6 m	4	—	Vs. placebo: ↓PWV, ↓TAG, leptin, and HOMA-IR, ↑adiponectin, ↓WC, visceral fat, and total body fat, ↓BMI and LDL-C, and ↑HDL-C (numerically, but not statistically significant)	[60]
*Curcuma longa* L./rhizome	Zingiberaceae	Turmeric	400 mg, TDS	Randomized, double-blind, placebo-controlled trial on diabetic patients treated with the plant (60) or placebo (54)	3 m	5	Metformin and sulfonylureas	Vs. placebo: ↓PWV, AI, and arterial stiffness and no significant effect on BP; vs. baseline: ↓ICAM-1 and VCAM-1	[61]
*Curcuma longa* L./rhizome	Zingiberaceae	Turmeric	700 mg, TDS	Randomized, double-blind, placebo-controlled trial on hyperlipidemic diabetic patients treated with the plant (36) or placebo (39)	2 m	5	Oral antidiabetics and antihyperlipidemics	Vs. placebo: ↓BMI, TAG, and TC, ↓LDL-C (borderline), no significant change in glycemic profile, apolipoproteins, and other lipid markers	[62]
*Cyamopsis tetragonoloba* (L.) Taub./partially hydrolyzed gum	Leguminosae	Guar	5 g, BD	Randomized, controlled clinical trial on diabetic patients with MetS treated with the plant (23) or left untreated (21)	1.5 m	3	Metformin and protamine insulin	Vs. baseline: ↓HbA1c, ↓serum *trans*-fatty acid, ↓WC, and no significant change in BP, lipid profile, CRP, and ET-1	[43]
*Cynara scolymus* L./leaf	Compositae	Artichoke	400 mg, TDS	Randomized, double-blind, placebo-controlled trial on diabetic hypercholesterolemic patients treated with the plant (36) or placebo (36)	2 m	4	Metformin and glyburide	Vs. placebo: no significant change in FBS, GTT, HbA1c, TAG, ↓TC, and LDL-C	[24]
*Cynara scolymus* L./petals	Compositae	Artichoke	6 g daily	Controlled trial on diabetic patients treated with the plant (15) or placebo (15)	3 m	—	—	Vs. baseline: ↓FBS and postprandial sugar, ↓TC, LDL-C, and TAG, and ↑HDL-C	[23]
*Dichrostachys glomerata* (Forssk.) Chiov./pod	Leguminosae	—	400 mg, BD	Randomized, double-blind, placebo-controlled trial on diabetic obese patients treated with the plant (23) or placebo (23)	2 m	2	ND	Vs. placebo: ↓FBS and HbA1c, ↓TC, LDL-C, TAG, and TC/HDL-C, ↑HDL-C, ↓SBP and DBP, and ↓BW, BMI, WC, HC, and body fat	[63]
*Fragaria × ananassa* (Duchesne ex Weston) Duchesne ex Rozier/fruit	Rosaceae	Strawberry	25 g freeze-dried powder, BD	Randomized, double-blind, placebo-controlled trial on diabetic patients treated with the plant (19) or placebo (17)	1.5 m	4	Oral antidiabetics	Vs. baseline and placebo: ↓HbA1c, CRP, and MDA, ↑TAOS, and no significant change in FBS and anthropometric parameters	[64]
*Ginkgo biloba* L./leaf	Ginkgoaceae	Ginkgo	1 tablet (containing flavonol glycoside 19.2 mg and terpene lactone 4.8 mg), TDS	Randomized, controlled trial on diabetic patients with nephropathy treated with the plant (32) or left untreated (32)	2 m	2	Oral antidiabetics, insulin, and antihypertensives	Vs. baseline: ↑NO, ↓VWF, and no significant change in FBS and ET-1	[42]
*Glycine max* (L.) Merr./bean	Leguminosae	Soy	2.5 g, daily	Controlled trial on diabetic hypercholesterolemic patients treated with fenofibrate (11), plant (18), or fenofibrate + plant (7)	2 m	—	Fenofibrate	Soybean + fenofibrate vs. fenofibrate alone: ↓LDL-C and TAG and no significant change in TC and HDL-C	[65]
*Glycine max* (L.) Merr.^*∗*^/bean (in the form of nut)	Leguminosae	Soy	60 g, daily	Randomized, controlled trial on patients treated with the plant (35) or placebo (35)	2 m	2	ND	Vs. baseline: ↓FBS, SBP, DBP, TC, LDL-C, and E-selectin and ↑TAOC and FMD; vs. control: ↓FBS, TC, LDL-C, and E-selectin, ↑TAOC, FMD, and no significant change in HDL-C and TAG	[66]
*Gymnema lactiferum* (L.) R.Br. ex Schult./leaf	Apocynaceae	Ceylon cow-tree	3.5 g, BD	Open-label trial on diabetic hypercholesterolemic patients treated with the plant (12) or placebo (14)	1 m	—	Oral antidiabetics	Vs. baseline: ↓FBS and HbA1c, ↓TC and LDL-C, and no significant change in HDL-C, TAG, and BW	[67]
*Juglans regia* L.^*∗*^/nut	Juglandaceae	Walnut	56 g/ day	Randomized, controlled, single-blind, crossover trial on 24 diabetic patients	2 m	2	—	Vs. baseline: ↑FBS, ↓TC and LDL-C, ↓BP in the control group, and no significant change in anthropometric parameters, HbA1c and HOMA-IR; vs. control: ↑FMD	[68]
*Juglans regia* L./leaf	Juglandaceae	Walnut	100 mg, BD	Randomized, double-blind, placebo-controlled trial on diabetic hypercholesterolemic patients treated with the plant (32) or placebo (29)	3 m	5	Oral antidiabetics	Vs. baseline: ↓FBS and HbA1c, ↓TC and TAG, and no significant change in LDL-C and HDL-C; vs. placebo: ↓FBS, ↓TAG, and no significant change in FPI and c-peptide	[69]
*Juglans regia* L./leaf	Juglandaceae	Walnut	100 mg daily for the first week and then 100 mg, BD	Randomized, double-blind, placebo-controlled trial on diabetic patients treated with the plant (20) or placebo (19)	2 m	5	Metformin and glibenclamide	Vs. baseline: ↓SBP, but not DBP, ↓BW and BMI, no significant change in lipid and glycemic profile, and ↓FBS and HbA1c in placebo	[70]
*Laurus nobilis* L./leaf	Lauraceae	Bay leaf	500 mg, QID	Randomized, placebo-controlled trial on diabetic patients treated with the plant (50) or placebo (15)	1 m	—	Oral antidiabetics	Vs. baseline: ↓FBS, ↓TC, LDL-C, and TAG, and ↑HDL-C	[71]
*Linum usitatissimum* L./seed gum	Linaceae	Flax	5 g daily (as chapatti)	Randomized, controlled trial on diabetic patients treated with the plant (60) or control chapattis (60)	3 m	2	ND	Vs. baseline: ↓FBS, ↓TC and LDL-C, and no significant change in VLDL-C, HDL-C, and TAG	[72]
*Lycopersicon esculentum* Mill.^*∗*^/fruit	Solanaceae	Tomato	250 ml, BD	Randomized, placebo-controlled trial on diabetic patients treated with the plant (15), vitamin E (12), vitamin C (12), or placebo (13)	1 m	3	Oral antidiabetics, ACE inhibitors	Vs. baseline: ↑LDL-C resistance to oxidation and no significant change in FBS, BP, CRP, and lipid profile	[73]
*Melissa officinalis* L./aerial parts	Lamiaceae	Lemon balm	350 mg, BD	Randomized, double-blind, placebo-controlled trial on diabetic patients treated with the plant (31) or placebo (31)	3 m	4	Oral antidiabetics	Vs. baseline: ↓ICAM-1 and TAG/HDL-C and ↑apo A-1; vs. placebo: ↓TC/HDL-C and LDL-C/HDL-C ↑apo A-1, and no significant change in ICAM-1	[74]
*Morus alba* L.^*∗*^/leaf	Moraceae	Mulberry	1 g, TDS	Randomized, double-blind, placebo-controlled pilot trial on diabetic patients treated with the plant (12) or placebo (12)	3 m	4	Oral antidiabetics	Vs. baseline and placebo: ↓postprandial blood glucose and no significant change in FBS, BW, BP, and HbA1c	[75]
*Panax ginseng* C.A.Mey./root	Araliaceae	Korean ginseng	100 mg, TDS	Randomized, double-blind, placebo-controlled trial on diabetic patients treated with the plant (20) or placebo (20)	2 m	4	ND	Vs. baseline and placebo: ↓IL-6 and CRP and no significant change in anthropometric parameters, TNF-*α,* and HbA1c	[30]
*Panax quinquefolius* L./root	Araliaceae	American ginseng	1 g, TDS	Randomized, double-blind, placebo-controlled trial on diabetic hypertensive patients treated with the plant (30) or placebo (34)	3 m	5	Oral antidiabetics	Vs. placebo: ↓radial AI and SBP, but not DBP, and no significant change in PP and HR	[37]
*Panax quinquefolius* L./root	Araliaceae	American ginseng	1 g, TDS	Randomized, double-blind, placebo-controlled, crossover trial on diabetic patients treated with the plant (24) or placebo (24)	2 m	5	Oral antidiabetics, Antihyperlipidemic agents, and antihypertensive drugs	Vs. placebo: ↓FBS and HbA1c, ↓SBP, ↓TC, LDL-C, TC/LDL-C, and LDL-C/HDL-C, ↑NOx, and no significant change in DBP; vs. baseline: ↓PAI-1	[76]
*Passiflora edulis* Sims/fruit peel	Passifloraceae	Purple passion fruit	220 mg, daily	Randomized, double-blind, placebo-controlled trial on diabetic patients treated with the plant (19) or placebo (21)	4 m	3	Metformin, glibenclamide, and atenolol	Vs. placebo: ↓FBS, ↓SBP, and no significant change in DBP, lipid profile, HbA1c, and BMI	[77]
*Phyllanthus emblica* L./fruit	Phyllanthaceae	Emblic	250 or 500 mg, BD	Randomized, double-blind, controlled trial on diabetic patients treated with low dose of the plant (20), high dose of the plant (20), atorvastatin (20), or placebo (20)	3 m	3	Oral antidiabetics	Vs. baseline and placebo: ↓HbA1c, ↓RI, ↓TC, LDL-C, and TAG, ↑HDL-C, ↓MDA and CRP, and ↑GSH and NO	[78]
*Pinus maritima* Mill./bark	Pinaceae	Pine	25 mg, 5 times a day	Randomized, double-blind, placebo-controlled trial on diabetic patients treated with the plant (24) or placebo (24)	3 m	3	Oral antidiabetics and /or antihypertensives	Vs. placebo: ↓FBS and HbA1c, ↓50% in ACE inhibitor dosage, and ↓LDL-C and ET-1	[79]
*Plantago ovata* Forssk./husk	Plantaginaceae	Psyllium	5 g, BD	Randomized, double-blind, placebo-controlled trial on diabetic patients treated with the plant (21) or placebo (15)	2 m	4	Oral antidiabetics	Vs. placebo: ↓FBS and HbA1c, ↓LDL-C/HDL-C, ↑HDL-C, and no significant change in FPI, TC, TAG, and LDL-C	[80]
*Portulaca oleracea* L./seed	Portulacaceae	Purslane	10 g, daily	Randomized, controlled, crossover trial on diabetic patients treated with the plant + yoghurt (48) or yoghurt alone (48)	5 weeks	1	Oral antidiabetics	Vs. baseline: ↓TC and TAG, ↓SBP and DBP, ↓BW, BMI, and WC, and no significant change in FPG, FPI, HOMA-IR, LDL-C, and HDL-C; vs. placebo: ↓TAG, ↓SBP, ↓BW and BMI, and no significant change in WC, FPG, FPI, HOMA-IR, TC, LDL-C, HDL-C, and DBP	[81]
*Portulaca oleracea* L./herb	Portulacaceae	Purslane	60 mg, TDS	Randomized, double-blind, placebo-controlled trial on diabetic patients treated with the plant (23) or placebo (27)	3 m	4	Oral antidiabetics	Vs. baseline: ↓SBP and no significant change in FBS and DBP	[82]
*Prunus amygdalus* Batsch^*∗*^/fruit	Rosaceae	Almond	56 g, daily	Randomized, crossover, controlled trial on diabetic hyperlipidemic patients treated with the plant (20) or placebo (20)	1 m	1	Oral antidiabetics	Vs. control: ↓ox-LDL-C, ↓IL-6, TNF-*α*, and CRP, and no significant change in MDA, ICAM-1, and VCAM-1	[83]
*Prunus amygdalus* Batsch^*∗*^/fruit	Rosaceae	Almond	56 g, daily	Randomized, crossover, controlled trial on diabetic hyperlipidemic patients treated with the plant (20) or placebo (20)	1 m	1	Oral antidiabetics	Vs. control: ↓FBS, FPI, and HOMA-IR ↓TC, LDL-C, LDL/HDL-C, apo B, apo B/ apo A-1, and nonesterified fatty acids, ↓body fat, and no significant change in BW and BMI	[84]
*Punica granatum* L.^*∗*^/fruit	Lythraceae	Pomegranate	200 ml, daily	Randomized, single-blind, controlled trial on diabetic patients treated with the plant (30) or left untreated (30)	1.5 m	3	ND	Vs. control: ↓ox-LDL-C and anti-ox-LDL-C antibody and ↑TAOS and PON-1	[34]
*Rheum ribes* L./stem	Polygonaceae	Syrian rhubarb	400 mg, TDS	Randomized, double-blind, placebo-controlled trial on diabetic hypercholesterolemic patients treated with the plant (18) or placebo (18)	1 m	3	Metformin and glyburide	Vs. placebo: ↓FBS, ↓TC and LDL-C, and no significant change in HDL-C and TAG	[85]
*Salvia miltiorrhiza* Bunge/root	Lamiaceae	Danshen	5 g, BD	Randomized, placebo-controlled trial on diabetic patients with coronary heart disease treated with the plant (31) or placebo (31)	2 m	2	Oral antidiabetics	Vs. placebo: ↓VCAM-1; vs. baseline: ↓VWF and ox-LDL-C	[41]
*Salvia officinalis* L./leaf	Lamiaceae	Sage	500 mg, TDS	Randomized, triple-blind, placebo-controlled trial on diabetic hyperlipidemic patients treated with the plant (40) or placebo (40)	3 m	5	Metformin and glyburide	Vs. baseline and placebo: ↓FBS and HbA1c, ↓TC, LDL-C, and TAG, and ↑HDL-C	[86]
*Satureja khuzestanica* Jamzad/aerial parts	Lamiaceae	—	250 mg, daily	Randomized, double-blind, placebo-controlled trial on hyperlipidemic diabetic patients treated with the plant (11) or placebo (10)	2 m	4	Oral antidiabetics and antihyperlipidemic agents	Vs. baseline: ↓TC and LDL-C, ↑HDL-C and TAOC, and no significant change in FBS, MDA, and TAG	[87]
*Sesamum indicum* L./seed	Pedaliaceae	Sesame	28 g, daily	Randomized, controlled trial on diabetic patients treated with the plant (20) or left untreated (16)	1.5 m	3	Oral antidiabetics	Vs. control: ↓TAG and AIP and no significant change in LDL-C, TC, and HDL-C	[88]
*Silybum marianum* (L.) Gaertn./seed	Compositae	Milk thistle	200 mg, TDS	Randomized, double-blind, placebo-controlled trial on hyperlipidemic diabetic patients treated with the plant (29) or placebo (25)	4 m	3	ND	Vs. baseline: ↓FBS, ↓TC, LDL-C, and TAG, and ↑HDL-C	[89]
*Silybum marianum* (L.) Gaertn./seed	Compositae	Milk thistle	140 mg, TDS	Randomized, triple-blind, placebo-controlled trial on diabetic patients treated with the plant (20) or placebo (20)	1.5 m	5	Oral antidiabetics	Vs. placebo: ↓MDA and CRP and ↑GSH, Gpx, SOD, and TAOS	[22]
*Silybum marianum* (L.) Gaertn./seed	Compositae	Milk thistle	140 mg, TDS	Randomized, triple-blind, placebo-controlled trial on diabetic patients treated with the plant (20) or placebo (20)	1.5 m	5	Oral antidiabetics	Vs. placebo: ↓FBS, FPI, and HOMA-IR, ↓QUICKI, ↓TAG and TAG/HDL-C, and ↑HDL-C; vs. baseline: ↓TC and LDL-C	[21]
*Thymus kotschyanus* Boiss. and Hohen./aerial parts	Lamiaceae	—	10 g, BD	Randomized, controlled trial on diabetic patients treated with the plant (32) or left untreated (32)	3 m	3	Oral antidiabetics	Vs. baseline: ↓FBS and HbA1c, ↓LDL-C, ↑HOMA-*β,* and no significant change in FPI, HOMA-IR, TC, and TAG; vs. control: ↑HDL-C	[90]
*Urtica dioica* L./aerial parts	Urticaceae	Stinging nettle	100 mg/kg	Randomized, single-blind, placebo-controlled clinical trial on diabetic patients treated with the plant (24) or placebo (21)	2 m	1	ND	Vs. baseline: ↓TNF-*α*; vs. placebo: ↓CRP and IL-6 and no significant change in FPI, insulin resistance, and anthropometric parameters	[32]
*Urtica dioica* L./aerial parts	Urticaceae	Stinging nettle	5 ml of liquid extract (containing 2.7 g/lit of dry matter), TDS	Randomized, double-blind, placebo-controlled trial on diabetic women treated with the plant (24) or placebo (23)	2 m	5	Metformin and glibenclamide	Vs. placebo: ↓FBS, TAG, and SGPT ↑HDL-C, NO, and SOD, and no significant change in other lipid profile parameters	[33]
*Vaccinium macrocarpon* Aiton^*∗*^/fruit	Ericaceae	Cranberry	500 mg, TDS	Randomized, double-blind, placebo-controlled trial on diabetic patients treated with the plant (15) or placebo (15)	3 m	2	ND	Vs. placebo: ↓TC, LDL-C, and TC/HDL-C, no significant change in ox-LDL-C, HDL-C, and TAG, and no significant change in BP, HbA1C, HOMA-IR, anthropometric parameters, and CRP	[91]
*Vaccinium macrocarpon* Aiton^*∗*^/fruit	Ericaceae	Cranberry	240 ml, daily	Randomized, double-blind, placebo-controlled trial on diabetic patients treated with the plant (29) or placebo (29)	3 m	3	Oral antidiabetics	Vs. baseline and placebo: ↓FBS, ↓apo-B, ↑apo-A-1 and PON-1 activity, and no significant change in Lp(a)	[35]
*Vitis* sp./seed	Vitaceae	Grape	300 mg, BD	Randomized, double-blind, crossover, placebo-controlled trial on 32 diabetic patients with high risk for cardiovascular problems	1 m	2	Oral antidiabetics or special diet control	Vs. baseline: ↓fructosamine and CRP, ↓TC, ↑GSH, and no significant change in HOMA, TAOS, and endothelial function	[36]
*Vitis vinifera* L./seed	Vitaceae	Grape	200 mg	Randomized, triple-blind, placebo-controlled trial on diabetic patients treated with the plant (26) or placebo (22)	2 m	3	—	↓HDL-C vs. baseline but not vs. placebo: no significant change in FBS, BP, TC, LDL-C, and TAG	[92]
*Zingiber officinale* Roscoe/rhizome	Zingiberaceae	Ginger	800 mg, BD	Randomized, double-blind, placebo-controlled trial on diabetic patients treated with the plant (33) or placebo (30)	3 m	4	—	Vs. placebo: ↓FBS, HbA1c, and HOMA-IR, ↓TC and TAG, ↑HDL-C/TC, and no significant change in HDL-C and LDL-C	[29]
*Zingiber officinale* Roscoe/rhizome	Zingiberaceae	Ginger	1 g, BD	Randomized, double-blind, placebo-controlled trial on diabetic patients treated with the plant (28) or placebo (30)	2 m	4	Oral antidiabetics and antihyperlipidemics	Vs. placebo: ↓FPI, ↓LDL-C, TAG, and HOMA-IR, ↑QUICKI, and no significant change in FPG, TC, HDL-C, and HbA1c	[28]
*Zingiber officinale* Roscoe/rhizome	Zingiberaceae	Ginger	1 g, TDS	Randomized, double-blind, placebo-controlled trial on diabetic patients treated with the plant (22) or placebo (23)	3 m	5	Oral antidiabetics	Vs. placebo: ↓FBS, FPI, HbA1c, and HOMA-IR, ↓CRP and MDA, and ↑TAOS and PON-1	[30]

Abbreviations: FBS: fasting blood sugar, TC: total cholesterol, LDL-C: low-density lipoprotein cholesterol, HDL-C: high-density lipoprotein cholesterol, TAG: triacyl glycerol, ADA: adenosine deaminase, CRP: C-reactive protein, HbA1c: glycosylated hemoglobin, AIP: atherogenic index of plasma, ox-LDL-C: oxidized LDL-C, BW: body weight, ASA: aspirin, MDA: malondialdehyde, GTT: 2-hour oral glucose tolerance test, TAOS: total antioxidant status, FMD: flow-mediated dilatation, ET: endothelin, AI: augmentation index, HR: heart rate, PP: pulse pressure, SBP: systolic blood pressure, DBP: diastolic blood pressure, IL: interleukin, TNF: tumor necrosis factor, PON-1: paraoxonase-1, ACE: angiotensin-converting enzyme, RI: reflection index, GSH: glutathione, SOD: superoxide dismutase, QUICKI: quantitative insulin sensitivity check index, ICAM: intracellular adhesion molecule, VCAM: vascular adhesion molecule, PAI: plasminogen activator inhibitor (the symbol ^*∗*^means the scientific name is not mentioned in the original article).

**Table 2:** Major possible mechanisms attributed to different phytochemical categories as protective agents in cardiovascular complications of type 2 diabetes mellitus.

Phytochemical categories	Possible mechanisms
Flavonoids, anthocyanins, and other polyphenols	Antioxidant, anti-inflammatory, and cytoprotective properties
Volatile terpenes and terpenoids	Regulation of high blood pressure via vasorelaxant effects
Nonvolatile terpenes and terpenoids	Anti-inflammatory, antihyperglycemic, antihypertensive, and vasculoprotective properties
Fibers	Slowing the dietary fat and sugar absorption and improvement of anthropometric parameters
Sulfated compounds	Cytoprotective effects on cardiomyocytes, antihypertensive and antihyperlipidemic effects, and anticoagulant activities
Alkaloids	Antihypertensive and vasorelaxant properties
